# Treatment outcomes of bedaquiline-resistant tuberculosis: a retrospective and matched cohort study

**DOI:** 10.1016/S1473-3099(25)00218-X

**Published:** 2025-10

**Authors:** Lindokuhle Mdlenyani, Zahraa Mohamed, Jacob A M Stadler, Nomfuneko Mtwa, Graeme Meintjes, Robin Warren, Matthew J Saunders, Johanna Kuhlin, Sean Wasserman

**Affiliations:** a Department of Health, Eastern Cape Province, East London, South Africa; b Wellcome Discovery Research Platforms in Infection, Centre for Infectious Diseases Research in Africa, Institute of Infectious Disease and Molecular Medicine, University of Cape Town, Observatory, Cape Town, South Africa; c Department of Medicine, University of Cape Town, Cape Town, South Africa; d Blizard Institute, Queen Mary University of London, London, UK; e Division of Molecular Biology and Human Genetics, SAMRC Centre for Tuberculosis Research, Faculty of Medicine and Health Sciences, Stellenbosch University, Cape Town, South Africa; f Institute for Infection and Immunity, City St George's, University of London, London, UK; g Department of Medicine Solna, Karolinska Institutet, Stockholm, Sweden; h Department of Infectious Diseases, Karolinska University Hospital, Stockholm, Sweden

## Abstract

**Background:**

Rising prevalence of bedaquiline resistance undermines benefits from this life-saving drug for rifampicin-resistant tuberculosis (RR tuberculosis). Despite increasing awareness, patient-level outcomes for bedaquiline-resistant tuberculosis have not been well characterised and case management has been poorly defined.

**Methods:**

We did a retrospective cohort study of bedaquiline-resistant tuberculosis with matched RR tuberculosis controls at a tuberculosis referral hospital in East London, South Africa. Cases included patients aged 13 years or older with a phenotypic bedaquiline-resistant *Mycobacterium tuberculosis* isolate identified between Jan 1, 2018 and June 30, 2023. Controls with confirmed bedaquiline-susceptible tuberculosis, matched 1:1 by baseline culture status, age, and HIV status, were selected from a prospective observational study conducted during an overlapping period at the same facility. Primary outcomes included time to sputum culture conversion (SCC), a modified WHO-defined unfavourable outcome, and tuberculosis-free survival (alive, with SCC, and in care or treatment completed) up until 18 months. Adjusted analyses used Cox proportional hazards and logistic regression models.

**Findings:**

82 patients with bedaquiline-resistant tuberculosis were included, 57 (70%) of whom were HIV positive. Bedaquiline was prescribed for 72 (88%) of 82 patients and meropenem (plus amoxicillin–clavulanate) for 32 (39%) of 82. Together with bedaquiline, the most frequently prescribed drugs included clofazimine, linezolid, and terizidone. Median time to SCC after treatment initiation was 175 days (IQR 100–254) in the bedaquiline-resistant cohort and 32 days (30–42) in matched controls. In the analysis of the combined cohorts, bedaquiline resistance (adjusted hazard ratio 0·03, 95% CI 0·0023–0·29, p=0·003) was associated with longer time to SCC when adjusted for baseline microscopy grade and baseline fluoroquinolone resistance. WHO treatment outcomes in those with bedaquiline-resistant tuberculosis were unfavourable in 54 (67%) of 81 patients, driven by treatment failure in 35 (43%) of 81. At 18 months, 43 (52%) of 82 patients had reached tuberculosis-free survival, 19 (23%) of 82 had died, and 50 (79%) of 63 survivors were still on treatment.

**Interpretation:**

Current treatment options for bedaquiline-resistant tuberculosis result in prolonged therapy, delayed microbiological responses, and poor clinical outcomes. Implementation of more rapid resistance testing, including targeted next-generation sequencing, and access to novel treatment options within randomised controlled trials for bedaquiline-resistant tuberculosis, are priorities for tuberculosis programmes.

**Funding:**

The South African Medical Research Council.

## Introduction

Bedaquiline has been transformational in improving outcomes and enabling treatment shortening for rifampicin-resistant tuberculosis (RR tuberculosis).^1^, ^2^, ^3^, ^4^ In 2023, an estimated 176 000 people were treated for RR tuberculosis, most of whom received bedaquiline as recommended by WHO and almost 60 national tuberculosis programmes (NTPs) had implemented the 6-month bedaquiline-linezolid-pretomanid (BPaL)-moxifloxacin regimen.^1^ The South African NTP has been providing bedaquiline to most people with RR tuberculosis since 2018 after its incorporation into a standardised 9–12-month oral regimen and current national guidelines recommend a BPaL-based regimen with levofloxacin instead of moxifloxacin.^5^, ^6^

Expanded use has been accompanied by emergence of bedaquiline resistance, which appears to be increasing.^1^ Resistance-associated genetic variants causing phenotypic resistance emerge during and after treatment, driven by subtherapeutic bedaquiline exposure due to its long elimination half-life.^7^, ^8^ These resistance mutants can be transmitted in communities.^9^ South African surveillance data from 2015 to 2019 found that 3·8% of pretreatment phenotypic bedaquiline resistance was strongly associated with previous bedaquiline exposure.^4^ More recently, bedaquiline resistance was detected among 3·6–10·2% of unselected patients with RR tuberculosis in South Africa^10^ and, in Mozambique, prevalence of genotypic bedaquiline resistance increased from 3% to 14% between 2016 and 2021.^11^ Bedaquiline resistance might worsen treatment outcomes, shown both in routine care^4^ and among patients from clinical trials evaluating BPaL,^12^ potentially undermining global effectiveness of shorter oral treatment for RR tuberculosis.

**Research in context**

**Evidence before this study**

We searched PubMed for reports on bedaquiline-resistant tuberculosis, management, and outcomes on Jan 25, 2025 from database inception using the key words ((“bedaquiline resistance”) OR (“BDQ resistance”)) AND (treatment) AND ((management) OR (outcomes)), filtering to include human studies only without language restrictions. We identified 24 articles: eight reviews, four case reports, four clinical studies without information on management or outcomes, three diagnostic or mechanistic studies, and one modelling study, leaving four articles describing treatment or treatment outcomes for people with bedaquiline-resistant tuberculosis. Three were individual patient studies (one prospective and two retrospective) and one was a population analysis using registry data from the South African National Treatment Programme (NTP). The three individual patient studies, conducted in South Africa, China, and Uzbekistan, included between five and 12 people with bedaquiline-resistant tuberculosis, although this was not uniformly defined, making treatment outcomes difficult to compare. Two studies reported inclusion of bedaquiline in treatment regimens, prescribed for five (42%) of 12 patients in the Uzbekistan study. Carbapenem therapy was provided to one (20%) of five patients and five (42%) of 12 in the South African and Uzbekistan cohorts, respectively. Treatment duration was not reported. By 6 months, between 40% and 92% of patients had achieved sputum culture conversion. Only the South African study reported end-of-treatment outcomes, which were unfavourable in three (60%) of five patients. Registry data from the South African NTP showed that a lower proportion of patients with bedaquiline resistance (73 [80%]) reached sputum culture conversion compared with those with bedaquiline-susceptible tuberculosis (1889 [87%]); time to sputum culture conversion was delayed in the bedaquiline-resistant group, but this difference was not statistically significant. Bedaquiline resistance was also associated with worse treatment outcomes, with treatment success in 57% (n=37) versus 72% (n=794) among those with bedaquiline-susceptible tuberculosis. No information on clinical management of bedaquiline resistance was available.

**Added value of this study**

Our study represents the largest published cohort describing clinical management and long-term treatment outcomes for people with bedaquiline-resistant tuberculosis. We found that, among a group of 82 people with confirmed bedaquiline-resistant tuberculosis, there was extensive resistance to other second-line antituberculosis agents. Despite treatment with a median of six drugs, including continued bedaquiline therapy in 72 (88%) and meropenem (plus amoxicillin-clavulanate) in 32 (39%), there was a much longer time to sputum culture conversion (175 days) compared with a matched cohort of patients with bedaquiline-susceptible tuberculosis (32 days) in the same programmatic setting. Only about half of cases with bedaquiline-resistant tuberculosis were alive and without a positive sputum culture for *Mycobacterium tuberculosis* and 50 (79%) of 63 survivors were still receiving treatment at 18 months after detection of bedaquiline resistance.

**Implications of all the available evidence**

Bedaquiline-resistant tuberculosis is an emergent phenomenon that threatens to undermine treatment of rifampicin-resistant tuberculosis and reverse progress in achieving the End TB goals set out by WHO. Our work adds to accumulating clinical observation that bedaquiline-resistant tuberculosis requires prolonged treatment with limited drug options, results in much longer time to sputum culture conversion, and is associated with poor treatment outcomes. This has important implications for tuberculosis programmes and the wider tuberculosis community. There is an urgent need to address bedaquiline resistance through clinical development of new treatment strategies, and to introduce rapid, near-patient resistance testing for bedaquiline (and companion drugs) to identify cases early, guide regimen selection, and reduce transmission.

Optimal treatment of bedaquiline-resistant tuberculosis is not known. WHO and the South African NTP recommend an individualised regimen guided by extended drug susceptibility testing (DST) and, in South Africa, consultation with a national clinical advisory committee.^6^, ^13^, ^14^ These so-called salvage regimens usually rely on the addition of WHO group C drugs including parenteral carbapenems.^15^ Reliance on bedaquiline-based treatment for RR tuberculosis, coupled with increasing population prevalence of bedaquiline resistance and scarce evidence to guide clinical management, requires improved understanding of the clinical effect of bedaquiline resistance. This study aimed to describe the management strategies and treatment outcomes of patients with bedaquiline-resistant pulmonary tuberculosis in a high HIV-burden setting in South Africa.

## Methods

### Study design and population

We did a retrospective cohort study of patients with bedaquiline-resistant RR tuberculosis, matched to controls with RR tuberculosis and confirmed bedaquiline susceptibility. All patients were treated within the South African NTP at Nkqubela Chest Hospital, a regional referral centre for drug-resistant tuberculosis in the Eastern Cape province, South Africa. Until March, 2023, the South African NTP performed bedaquiline phenotypic DST for selected RR tuberculosis cases, including those with poor treatment responses or known resistance to fluoroquinolones or isoniazid. After March, 2023, all *Mycobacterium tuberculosis* isolates from RR tuberculosis cases were tested.^4^, ^10^

The retrospective cohort included patients aged 13 years or older diagnosed with sputum culture-positive pulmonary tuberculosis and found to have a bedaquiline-resistant *M tuberculosis* isolate on phenotypic DST between Jan 1, 2018 and June 30, 2023. The date range was selected to identify cases after established bedaquiline use in the NTP and to capture 18-month follow-up information. Individuals without available treatment information were excluded.

The control group was selected from a prospective observational study (SHIFT-TB, n=260) that evaluated programmatic outcomes with an oral bedaquiline-based 9–12-month regimen at the same facility and received treatment within an overlapping time period (Jan 1, 2021 to Aug 30, 2022).^16^ Controls were matched 1:1 with bedaquiline-resistant patients on age, HIV status, and baseline culture status (appendix p 2). No participants enrolled in the SHIFT-TB study had previous RR tuberculosis treatment because this was an exclusion criterion for the regimen being evaluated. The matched cohort was included to provide internal validity and to contrast treatment-related and clinical outcome measures with a typical group of patients treated for RR tuberculosis in the national programme.

In the SHIFT-TB cohort, written informed consent or assent and parental consent for participants younger than 18 years was sought before participation in the study. Given that we used routinely collected data for the bedaquiline-resistant cohort, a waiver for consent and assent was approved by ethics committees.

Ethical approval was obtained from the Human Research Ethics Committee, University of Cape Town (887/2023 and 690/2019) and the Eastern Cape Department of Health Ethics Committee (EC_202311_022 and EC_201911_017).

### Procedures

We reviewed hospital files, pharmacy records, the National Health Laboratory Service (NHLS) database, and EDRWeb for patients in the bedaquiline-resistant cohort. For matched controls, we extracted data from the SHIFT-TB database. Information was captured until treatment completion, 18 months follow-up time, or study end (Jan 20, 2025), whichever was later (appendix p 2). Data were entered into a Research Electronic Data Capture database (Vanderbilt University, Nashville, TN, USA).

All microbiological samples for *M tuberculosis* were processed by the NHLS, including routine testing with rapid molecular tests and culture using Mycobacterial Growth Indicator Tube (MGIT; Becton and Dickson, Franklin Lakes, NJ, USA).^17^ Bedaquiline phenotypic DST was done using MGIT at the NHLS and at the Division of Molecular Biology and Human Genetics, Stellenbosch University, Cape Town, South Africa. A critical concentration of 1 mg/L was used^18^ with quality control carried out including *M tuberculosis* H37Rv daily (NHLS) or on each new test batch (Stellenbosch University).

### Outcomes and definitions

We analysed three main treatment outcomes: time to sputum culture conversion (SCC); a modified WHO-defined unfavourable outcome; and tuberculosis-free survival.

SCC was defined as the date of the first of two sputum samples with negative *M tuberculosis* cultures, consecutive or not, without any intervening positive culture. Reversion included any single positive culture following SCC. Our definition of SCC was less stringent than the 2021 WHO definition, considering less frequent sputum collection when using data from routine care in the bedaquiline-resistant cohort.^19^

We measured time to SCC up to 12 months from treatment initiation, defined relative to the date of collection of the first bedaquiline-resistant *M tuberculosis* isolate (referred to as the index sputum). Date of treatment initiation was assigned as the index sputum date for patients receiving treatment before the index sputum, or the treatment start date for patients not yet on treatment at the index sputum. Time to first SCC was defined as the time to the first occurrence of conversion, irrespective of subsequent reversion. Time to sustained SCC considered SCC only if it was maintained without reversion. This distinction accounted for patients with fluctuating culture status, with sustained SCC reflecting stable conversion.

Unfavourable treatment outcome was adapted from the WHO 2021 definition, which includes permanent regimen change for any reason, loss to follow-up, or death (appendix p 2).^13^ Because there is no defined treatment duration for bedaquiline-resistant tuberculosis, we used a patient-specific endpoint of treatment cessation for at least 2 months for outcome reporting.

Tuberculosis-free survival was defined as a composite of sustained SCC, being alive, and either having completed treatment or being in care for tuberculosis.

### Statistical analysis

Clinical characteristics were described using summary statistics and individual patient treatment trajectories were visually presented. Incomplete data were handled using a complete case approach if less than 10% were missing (appendix p 2).

Cox proportional hazards were estimated for time to sustained SCC in the bedaquiline-resistant cohort, and the following potential predictors were evaluated: age; sex; BMI; HIV and antiretroviral status; CD4 count; baseline microscopy positivity; and previous treatment for RR tuberculosis. Bedaquiline use, from treatment initiation, was included as a binary variable because of bidirectional causation. For variables of interest that did not meet the proportional hazards assumption, we estimated Kaplan–Meier survival curves stratified by the relevant variable.

Matched participants from SHIFT-TB (unexposed group) were pooled with the bedaquiline-resistant cohort (exposed group) and a stratified Cox regression model was used to account for matching. Cox proportional hazards were estimated for time to sustained SCC with the primary exposure variable being bedaquiline resistance, adjusting for potential predictors of sex, BMI, baseline microscopy grade, and baseline fluoroquinolone resistance. Previous RR tuberculosis treatment could not be assessed given that none of the controls had previously been treated for RR tuberculosis. Time to SCC was quantified by restricted mean survival time over 12 months from treatment start. Kaplan–Meier estimates, stratified by bedaquiline resistance, were compared with the log-rank test.

Predictors of unfavourable tuberculosis-free survival at 18 months for the bedaquiline-resistant cohort were explored with logistic regression, testing the aforementioned predictors: age; sex; BMI; HIV and antiretroviral status; CD4 count; baseline microscopy positivity; previous treatment for RR tuberculosis; and duration of bedaquiline treatment plus meropenem use. Following peer review we also evaluated the effect of individual drugs and number of drugs at treatment initiation.

A secondary analysis of time to death was done using a similar strategy as time to sustained SCC, except that bedaquiline duration (months) was included as a continuous predictor. To mitigate immortal time bias in the bedaquiline-resistant group, a sensitivity analysis included only patients who survived at least 8 weeks after the index sputum.

Forward selection of predictors for multivariable analyses was based on a univariable p value lower than 0·15. Deviations from the original protocol are described in the appendix (p 3). All statistical analysis was done using RStudio version 2024.09.0+375.

### Role of the funding source

The funder of the study had no role in study design, data collection, data analysis, data interpretation, or writing of the report.

## Results

We identified 86 patients with bedaquiline-resistant pulmonary tuberculosis, 82 of whom were included in the analysis. Reasons for exclusion were missing hospital records (n=2) and treatment at a different institution (n=2). Characteristics of the bedaquiline-resistant cohort and matched bedaquiline-susceptible controls were similar, besides higher baseline microscopy positivity, no previous RR tuberculosis, lower fluoroquinolone resistance (seven [9%] of 82 *vs* 70 [85%] of 82), and more advanced HIV disease in the bedaquiline-susceptible cohort. Most patients in the bedaquiline-resistant cohort had previous exposure to bedaquiline or clofazimine (table 1).

**Table 1:** Demographic and clinical characteristics of patients with bedaquiline-resistant tuberculosis at the time of index sputum collection and bedaquiline-susceptible controls at treatment start

		**Bedaquiline-resistant group (n=82)**	**Bedaquiline-susceptible group (n=82)**
Age, years			
	Median (Q1–Q3)	38 (30–46)	37 (32–44)
	Median (minimum–maximum)	38 (14–74)	37 (18–61)
Sex			
	Female, reported by health-care worker	42 (51%)	37 (45%)
	Male, reported by health-care worker	40 (49%)	45 (55%)
Weight, kg			
	Median (Q1–Q3)	52 (44–61)	52 (46–60)
	Median (minimum–maximum)	52 (33–96)	52 (35–97)
BMI*, kg/m^2^			
	Median (Q1–Q3)	18·4 (16·8–21·7)	18·2 (16·6–21·5)
	Median (minimum–maximum)	18·4 (13·4–35·4)	18·2 (12·0–38·4)
Sputum microscopy positive		45/81 (56%)	59 (72%)
Baseline microscopy grade			
	0	36/81 (44%)	23 (28%)
	>1	20/81 (25%)	18 (22%)
	>2	14/81 (17%)	20 (24%)
	>3	11/81 (14%)	21 (26%)
HIV test positive		57/81 (70%)	57 (70%)
ART status (if HIV positive)			
	Currently on ART	51 (89%)	32 (56%)
	Not on ART	6 (11%)	25 (44%)
HIV viral load† (if HIV positive)‡			
	Below level of detection	13 (33%)	3 (7%) of 42
	Detectable	27 (68%)	39 (93%) of 42
Quantitative viral load†			
	Median (Q1–Q3)	6600 (1200–117 600)§	42 737 (226–592 528)¶
	Median (minimum–maximum)	6600 (100–3 520 000)§	42 737 (26–2 538 616)¶
CD4 count†, cells per mm^3^			
	Median (Q1–Q3)	167 (82–335)‖	100 (27–210)**
	Median (minimum–maximum)	167 (19–1170)‖	100 (2–682)**
Number of medications at treatment initiation			
	Median (Q1–Q3)	6 (5–7)	7 (7–7)
	Median (minimum–maximum)	6 (3–8)	7 (5–7)
Previous RR-tuberculosis episodes			
	0	42 (51%)	82 (100%)
	≥1	40 (49%)	0
Previous RR-tuberculosis outcome loss to follow-up		15 (18%)	Not applicable
Previous RR-tuberculosis outcome treatment failure		22 (27%)	Not applicable
Previous treatment with bedaquiline or clofazimine		65 (79%)	0
	Previous treatment with bedaquiline	64 (78%)	0
	Previous treatment with clofazimine	65 (79%)	0

Index sputum is defined as the first bedaquiline-resistant *Mycobacterium tuberculosis* isolate. ART=antiretroviral treatment. Q=quartile. RR=rifampicin-resistant.

* n=81.

† Within 6 months before and after the time of collection of the bedaquiline-resistant sputum.

‡ n=40 for the bedaquiline-resistant group and n=42 for the bedaquiline-susceptible group.

§ n=27.

¶ n=39 had detectable viral loads.

‖ n=40.

** n=49.

At the time of index sputum, 70 (85%) of 82 patients fulfilled criteria for extensively drug-resistant tuberculosis (resistance to both bedaquiline and fluoroquinolones),^13^ two of whom (two (3%) of 68) had additional resistance to linezolid (appendix p 5). Of the *M tuberculosis* isolates tested after the index sputum, seven (21%) of 33 had linezolid resistance, all obtained from patients who had a previous susceptible result. Phenotypic clofazimine resistance was present in 67 (92%) of the 73 baseline isolates tested. 19 (95%) of 20 patients who had bedaquiline DST done before the index sputum date had bedaquiline-susceptible results (the patient with a previous resistant isolate was treated at a different institution during that episode).

**Figure 1:**
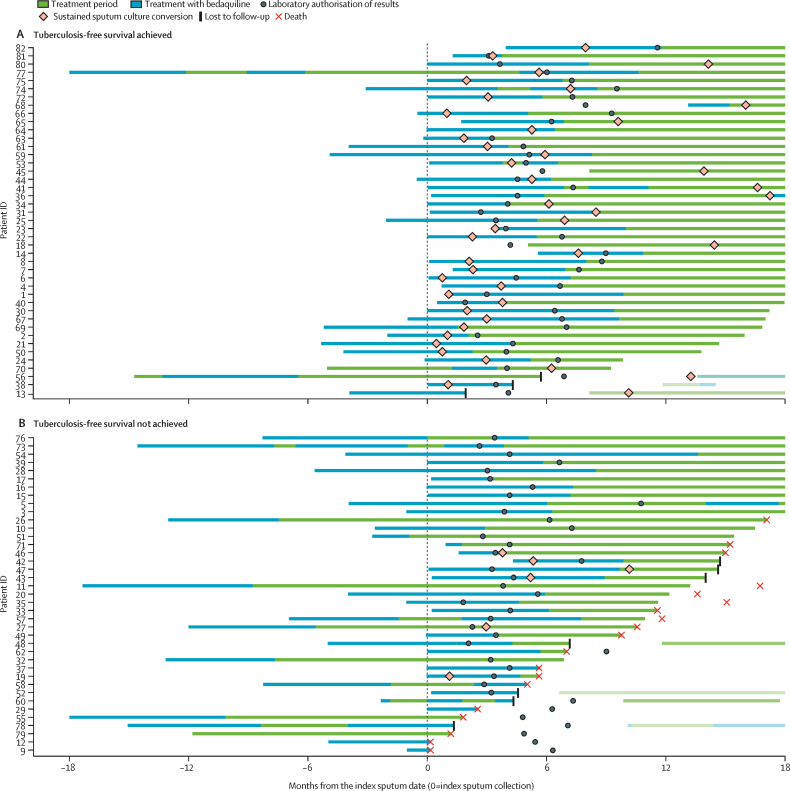
Treatment timeline for patients with bedaquiline-resistant tuberculosis Ordered by treatment end date within each outcome group. Faded lines represent subsequent treatment episodes with with drugs other than bedaquiline.

**Figure 2:**
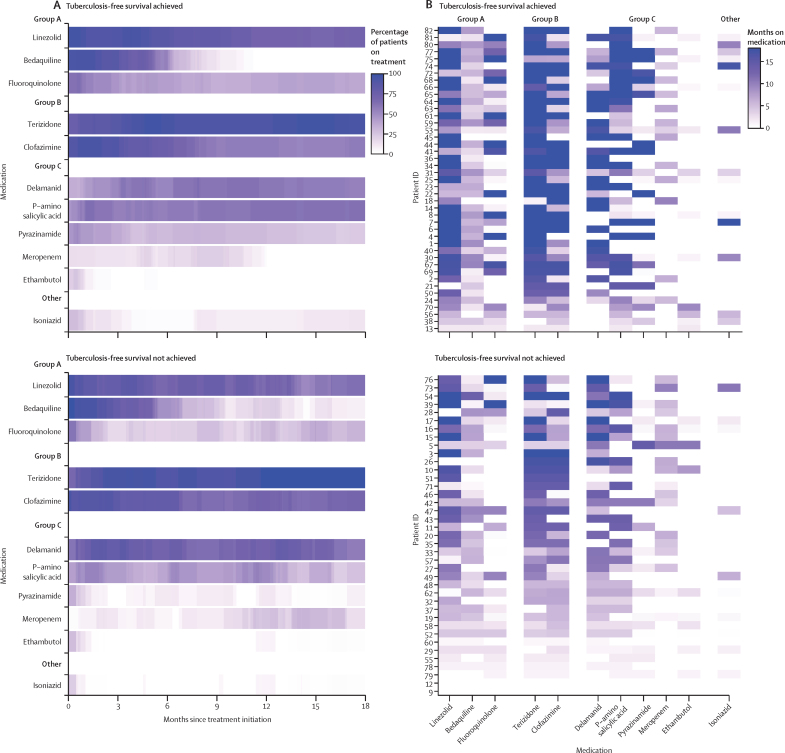
Antituberculosis therapy for bedaquiline-resistant tuberculosis (A) Proportion of patients prescribed each drug over time. (B) Duration of individual drugs prescribed per patient. Censored for death, loss to follow-up, and at 18 months. Fluoroquinolone is levofloxacin or moxifloxacin. Ethionamide (prescribed for six patients) and rifabutin (prescribed for four patients) were omitted for readability.

Laboratory reporting of the bedaquiline-resistant result occurred at a median of 4·5 months (IQR 3·4–6·7) after index sputum collection, during which time patients continued receiving treatment. Treatment modification occurred in 26 (59%) of 44 patients and sustained SCC was reached in 28 (57%) of 49 by the time bedaquiline-resistant results were reported. Median overall treatment duration after the index sputum was 17·7 months (IQR 10·4–20·4). Bedaquiline was included at some point for 72 (88%) of 82 patients, continued after the laboratory report of resistance in 48 (68%) of 71 patients for a median of 65 days (IQR 39–138), and provided for an overall median duration of 5·5 months (range 4 days to 13·5 months) after the index sputum (figure 1; appendix p 6). Treatment regimens varied substantially in composition and duration (figure 2). Together with bedaquiline, the most frequently prescribed drugs included clofazimine, linezolid, and terizidone. No patients were prescribed amikacin despite susceptibility in some cases. Meropenem (plus amoxicillin-clavulanate) was included in the regimen for 32 (39%) of 82 patients and started at a median 156 days (IQR 115–236) after the index sputum (figure 2; appendix p 6).

**Figure 3:**
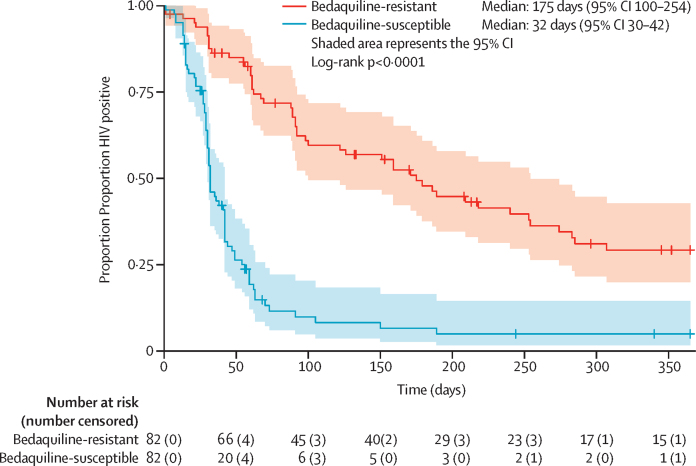
Kaplan–Meier curve for sustained sputum culture conversion after treatment initiation in the combined cohort of bedaquiline-resistant cases and matched bedaquiline-susceptible controls Censored at death, lost to follow-up, and at 12 months.

In the bedaquiline-resistant group, median time to first SCC was 100 days (95% CI 89–179; appendix p 15) and time to sustained SCC was 175 days (100–254; figure 3). None of the variables tested were significantly associated with sustained SCC (appendix p 7). Therefore, a multivariable analysis was not conducted.

Median time to sustained SCC was 32 days (95% CI 30–42) in the bedaquiline-susceptible cohort, 137 days (102–172; log rank p<0·0001) earlier than in the bedaquiline-resistant cohort (figure 3). Time to sustained SCC in the bedaquiline-susceptible cohort was similar after removing additional sputum samples collected as part of the observational study protocol (appendix p 4). In the pooled analysis of bedaquiline-resistant patients and matched bedaquiline-susceptible controls, bedaquiline resistance was associated with longer time to sustained SCC (adjusted hazard ratio 0·03, 95% CI 0·0023–0·29, p=0·003), after adjustment for baseline microscopy grade and baseline fluoroquinolone resistance (appendix p 8).

Treatment outcome according to the modified WHO definition measured at the end of treatment was unfavourable in 54 (67%) of 81 patients with bedaquiline-resistant tuberculosis (appendix p 9). Treatment failure, driven by an absence of culture conversion by 6 months, accounted for unfavourable outcomes in 35 (43%) of 81 patients and eight (10%) of 81 were lost to follow-up.

Tuberculosis-free survival after the index sputum was reached at 6 months for 41 (50%) of 82 patients, 12 months for 47 (57%) of 82 patients, and 18 months for 43 (52%) of 82 patients with bedaquiline-resistant tuberculosis (table 2). The reduction in tuberculosis-free survival between month 12 and month 18 was explained by worse outcomes among nine patients in that interval, five (56%) of whom culture reverted, three (33%) of whom were lost to follow-up, and one (11%) of whom died. 50 (79%) of 63 survivors were still on treatment at 18 months. At 6 months, eight (10%) of 82 patients had died, increasing to 19 (23%) of 82 at 18 months. Results were similar when assessed from treatment initiation following the index sputum (appendix p 10). There was an association between fewer prescribed drugs at treatment initiation (adjusted odds ratio [aOR] 0·66, 95% CI 0·43–0·97, p=0·042), and a possible association between fewer months of bedaquiline use (aOR 0·88, 0·75–1·02, p=0·10), and unfavourable tuberculosis-free survival at 18 months (appendix p 11). Tuberculosis-free survival for the bedaquiline-susceptible group is reported in the appendix (p 12).

**Table 2:** Tuberculosis-free survival at 6 months, 12 months, and 18 months after index sputum collection in patients with bedaquiline-resistant tuberculosis

			**Month 6 (n=82)**	**Month 12 (n=82)**	**Month 18 (n=82)**
Outcome reached			41 (50%)	47 (57%)	43 (52%)
	Alive, tuberculosis free, and treatment complete*		0	2 (2%)	8 (10%)
	Alive, tuberculosis free, and in care (treatment ongoing)		41 (50%)	45 (55%)	35 (43%)
Outcome not reached			41 (50%)	35 (43%)	39 (48%)
	Died		8 (10%)	13 (16%)	19 (23%)
	Not tuberculosis free, alive, and in care (treatment ongoing)		26 (32%)	18 (22%)	15 (18%)
	Not in care†		7 (9%)	4 (5%)	5 (6%)
		Alive, tuberculosis free	2 (2%)	1 (1%)	3 (4%)
		Alive, not tuberculosis free	5 (6%)	3 (4%)	2 (2%)

Index sputum is defined as the first bedaquiline-resistant *Mycobacterium tuberculosis* isolate.

* Tuberculosis free is defined as reaching sputum culture conversion without culture reversion by the specified timepoint.

† Loss to follow-up (irrespective of tuberculosis status) or completed treatment but not tuberculosis free.

**Figure 4:**
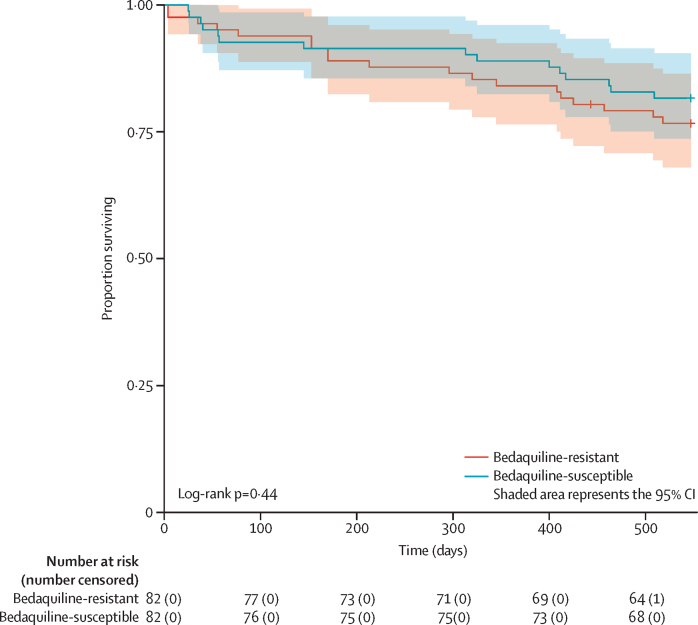
Kaplan–Meier curve for survival in the combined cohort of bedaquiline-resistant cases and matched bedaquiline-susceptible controls from index sputum collection Censored at 18 months.

Longer duration of bedaquiline use among patients with bedaquiline-resistant tuberculosis was associated with reduced mortality over 18 months (adjusted hazards ratio 0·74, 95% CI 0·62–0·88, p=0·0008; appendix p 13). This association was maintained on sensitivity analysis including only patients who survived at least 8 weeks after collection of the index sputum (appendix p 14). Use of meropenem was not associated with survival (appendix p 16). A Cox regression analysis combining the bedaquiline-resistant and bedaquiline-susceptible cohorts could not be done because of non-proportional hazards. Cumulative mortality at 18 months was numerically higher at 22% (95% CI 13–31) in the bedaquiline-resistant cohort versus 18% (9–26) in the bedaquiline-susceptible cohort, but this was not statistically significant (figure 4; log-rank test p=0·45).

## Discussion

Our study characterised management and long-term outcomes among patients treated for bedaquiline-resistant tuberculosis in the South African NTP. Only half of patients were alive and tuberculosis free at 18 months after detection of bedaquiline resistance, and time to SCC was almost 5 months longer than a matched RR tuberculosis cohort without bedaquiline resistance. Treatment outcomes are similar to those of extensively drug-resistant tuberculosis (previously defined as resistance to an injectable drug and fluoroquinolones) in the pre-bedaquiline era,^20^, ^21^ highlighting the severe consequences of bedaquiline resistance to patients and tuberculosis programmes.

Poor microbiological treatment responses, indicated by longer time to SCC among bedaquiline-resistant patients compared with matched controls, reinforces the need for an active bactericidal agent in regimens for bedaquiline-resistant tuberculosis, even when other effective group-A and group-B drugs such as linezolid and terizidone are included. Patients with bedaquiline-resistant tuberculosis in our study had worse WHO treatment outcomes (33% favourable treatment outcome) compared with the most recent global estimates for RR tuberculosis (68%)^1^ and those from a retrospective study in the South African NTP of unselected patients with RR tuberculosis on bedaquiline-containing regimens (67%).^3^ In our study, these outcomes were driven by failure to culture convert and culture reversions, again highlighting the reduced effectiveness of RR tuberculosis regimens with impaired or absent bedaquiline efficacy. An additional factor is the more extensive resistance profiles in bedaquiline-resistant isolates leading to a lower overall number of effective drugs.^12^, ^22^ Most *M tuberculosis* isolates from patients in our study were resistant to key second-line drugs, including fluoroquinolones and clofazimine (>90% resistant).

In a recent analysis of programmatic data from the South African NTP, end of treatment mortality for RR tuberculosis with shorter bedaquiline-based regimens was around 17%, increasing over time to 24% at 24 months.^10^, ^23^ Similar mortality trends were seen among people with bedaquiline-resistant tuberculosis in our study. Cause of death was not ascertained, but presumably a substantial number of people experienced late mortality from uncontrolled tuberculosis disease, given that almost 20% of survivors were culture positive for *M tuberculosis* at 18 months despite treatment. Other potential explanations for the high mortality include advanced HIV disease (more than two-thirds were HIV-positive with a median CD4 count of 167 cells per mm^3^) and functional lung damage (half of cases had previous episodes of RR tuberculosis and over a quarter experienced previous treatment failure). We were not able to access radiological data and, therefore, could not assess the extent of pulmonary disease in both cohorts, which might have influenced outcomes.

Bedaquiline use is associated with mortality reduction in RR tuberculosis^24^ and resistance is therefore expected to reduce this effect. In our study, cumulative mortality was numerically higher among patients with bedaquiline resistance than in controls. Survival bias might artificially lower mortality estimates because patients might die before undergoing resistance testing for bedaquiline, possibly explaining our results. Another potential explanation for attenuated mortality is residual treatment effect. We found an association between duration of bedaquiline use and reduced mortality. Although this observation could be partially explained by immortal time bias, bedaquiline might have an antituberculosis effect against bedaquiline-resistant variants with moderate minimum-inhibitory-concentration (MIC) elevations. The clinical efficacy of this strategy is unknown^25^, ^26^ and MIC data were unavailable in our study.

Extensive use of bedaquiline in our cohort is not aligned with South African treatment guidelines, which recommend use of group-C drugs, and not continuation of bedaquiline, in cases of resistance. We hypothesise several contributing factors from our data. First, the long delay (median 4·5 months) between collection of the index sputum and laboratory release of results, plus absence of an automated feedback system, might limit clinician awareness. Second, clinicians might decide to continue bedaquiline while awaiting a confirmed resistance result or in the context of confirmed resistance, particularly among stable patients or in those showing clinical improvement. In our study, SCC occurred in 57% of cases before bedaquiline resistance was reported and several patients were successfully treated with continuation of bedaquiline-containing regimens.

Fewer than half of patients in our cohort were prescribed a carbapenem despite inclusion in national and WHO guideline recommendations as part of salvage regimens for highly resistant tuberculosis.^6^ Carbapenem use in drug-resistant tuberculosis has been informed by observational studies that focused on difficult-to-treat cases (treatment success 57–80%),^27^ and an individual patient meta-analysis showing reduced treatment failure or relapse (aOR 0·4, 95% CI 0·2–0·7).^13^, ^28^ Meropenem exposure in the bedaquiline-resistant group was not associated with improved outcomes, although this is limited by confounding by indication and potential allocation bias. We did not ascertain reasons for treatment decisions to explain relatively low meropenem use. Plausible explanations are reluctance for intravenous therapy by caregivers and patients, favourable clinical responses on continued bedaquiline therapy, and mortality occurring before meropenem could be started (higher early mortality was observed among those not receiving meropenem; appendix p 16).

The long delay between index sputum collection and treatment initiation and prolonged time to SCC may contribute to community transmission, accelerating the public health threat of bedaquiline resistance. Of concern is the increasing proportion of patients in our cohort with *M tuberculosis* isolates resistant to linezolid (from 3% to 21%), implying an additional risk of linezolid resistance amplification. Implementation of near-patient and easily interpretable rapid molecular testing, such as commercial assays for targeted next-generation sequencing, for detecting bedaquiline resistance (and for companion drugs) is a priority for tuberculosis programmes, and is being rolled out by the South African NTP.^10^ Additional strategies being considered include early identification of patients who disengage from care on bedaquiline-based regimens and contact tracing to reduce transmission and identify hotspots.^10^

Our study had limitations. Patients were identified from a single treatment centre, affecting generalisability because of differences in clinical management, host factors (eg, HIV prevalence), and infecting *M tuberculosis* strains. Systematic error in inclusion might have occurred since patients with bedaquiline-resistant tuberculosis improving on treatment might not have been treated at our study site. However, only two patients were excluded for this reason. The matched analysis is prone to random error because of the small sample size determined by restrictive matching criteria. Although we matched on important prognostic factors, the comparison is imperfect because of residual differences in the populations that might confound outcomes (eg, lower fluoroquinolone resistance, previous treatment episodes, and differing frequency of sputum collection in the control group). Timing of treatment directed towards bedaquiline-resistant tuberculosis was unreliably documented in medical records. We therefore selected time of index sputum collection for analysis of the key clinical outcomes to reflect real-world experience and avoid survival bias associated with delays in treatment start. During the study period, bedaquiline-resistance testing was mainly offered to patients with poor treatment response or fluoroquinolone resistance. This approach might bias towards a sicker population, overestimating the effect of bedaquiline resistance on tuberculosis treatment outcomes. The South African NTP now recommends routine bedaquiline DST for all RR tuberculosis cases and continued surveillance is necessary to determine more representative outcomes.^6^ Finally, BPaL-based regimens, now recommended as first line for RR tuberculosis, were not yet implemented during our study period and it is unknown how widespread use might influence dynamics and clinical impact of bedaquiline resistance.

In conclusion, our study shows that people with bedaquiline-resistant tuberculosis have limited treatment options and, consequently, suffer poor treatment outcomes despite prolonged antituberculosis therapy with available drugs.

### Contributors

### Data sharing

Individual deidentified patient data from the bedaquiline-resistant cohort and the control group, including a data dictionary defining each variable in the set, are available upon request to the corresponding author after approval of study proposal and a signed data-sharing agreement.

## Declaration of interests

GM was an independent member of the drug safety monitoring board for the Bill & Melinda Gates Medical Research Institute and for Otsuka Pharmaceutical trials. RW was employed by the South African Medical Research Council who partially funded the study. SW received grants from the Bill & Melinda Gates Foundation and the National Institutes of Health, USA. All other authors declare no competing interests.
