# Supplementary material for: Treatment outcomes of bedaquiline-resistant tuberculosis: a retrospective and matched cohort study
Source: Lancet Infect Dis. 2025 Oct;25(10):1149–58. doi: 10.1016/S1473-3099(25)00218-X (PMC12662777; doi:10.1016/S1473-3099(25)00218-X)
Supplement: Supplementary appendix [file mmc1.pdf]

THE LANCET
Infectious Diseases

Supplementary appendix

This appendix formed part of the original submission and has been peer reviewed. We post it as supplied by the authors.

Supplement to: Mdlenyani L, Mohamed Z, Stadler JAM, et al. Treatment outcomes of bedaquiline-resistant tuberculosis: a retrospective and matched cohort study. *Lancet Infect Dis* 2025; published online June 18. https://doi.org/10.1016/S1473-3099(25)00218-X.

APPENDIX

Supplementary Methods 2

Supplement Table 1: Protocol deviations 3

Supplementary Results 4

Supplement Table 2: Drug susceptibility testing of sputum and proportion with resistance to each drug before, at, and after the index sputum[1] collection when bedaquiline resistance was identified 5

Supplement Table 3: Treatment regimens and treatment duration from treatment initiation[1] after the index sputum during which bedaquiline resistance was identified 6

Supplement Table 4: Sustained sputum culture conversion over 12 months after treatment initiation[1] in patients with bedaquiline-resistant tuberculosis using a Cox proportional hazards model 7

Supplement Table 5: Time to sustained sputum culture conversion over 12 months after treatment initiation in bedaquiline-resistant cases and matched bedaquiline-susceptible controls using a stratified Cox proportional hazard model 8

Supplement Table 6: Modified World Health Organization treatment outcomes after treatment initiation[1] in patients with bedaquiline-resistant tuberculosis 9

Supplement Table 7: Tuberculosis-free survival at 6, 12, and 18 months after treatment initiation[1] in patients with bedaquiline-resistant tuberculosis 10

Supplement Table 8: Unfavourable tuberculosis-free survival at 18 months after treatment initiation in patients with bedaquiline-resistant tuberculosis using a logistic regression model 11

Supplement Table 9: Tuberculosis-free survival at 6, 12, and 18 months after treatment start in the control group with bedaquiline-susceptible tuberculosis 12

Supplement Table 10: Time to death over 18 months from index sputum[1] collection in patients with bedaquiline-resistant tuberculosis using a Cox proportional hazards model 13

Supplement Table 11: Time to death over 18 months from index sputum[1] in patients with bedaquiline-resistant tuberculosis who survived at least eight weeks using a Cox proportional hazards model (sensitivity analysis) 14

Supplement Figure 1: Time to first sputum culture conversion from treatment initiation in bedaquiline-resistant cases and matched bedaquiline-susceptible controls until 12 months, by bedaquiline resistance 15

Supplement Figure 2: Survival from time of index sputum[1] collection in patients with bedaquiline resistant tuberculosis until 18 months, by meropenem use 16

References 17

Supplementary Methods

Controls with confirmed phenotypic bedaquiline susceptibility were matched to bedaquiline-resistant cases based on age, HIV status, and baseline sputum culture status. Age matching was conducted by categorising patients into the following age groups: 10–20 years, 20–30 years, 30–40 years, 40–50 years, 50–60 years, and over 60 years. When two or more controls were found for a case, one was picked randomly using the slice_sample function in the dplyr package in RStudio Version 2024·09·0+375 (Posit team, Boston, MA, USA).

For the bedaquiline-resistant cohort we used multiple sources for data collection, including hospital records, the Electronic Drug-Resistant Tuberculosis Register (EDR-Web), the National Health Laboratory Service (NHLS) records, and the South African Population Register. Data collection included demographics, comorbidities, microbiological data, previous tuberculosis (TB) treatment, antituberculosis medication, treatment outcomes, and vital status until end of treatment, 18 months follow-up time, or study end (20 January 2025). Where possible, multiple records from different data sources were used to confirm data validity (e.g. both the EDRWeb and South African Population Register were used to confirm vital status). For the matched controls, clinical information was obtained directly from study participants during study visits, but other data sources were the same as for the bedaquiline-resistant cohort.

We used a complete case analysis if <10% of data was missing and included a "missing" category if 10% or more was missing to reduce bias. Overall, there were few missing data points for the main analyses. In the bedaquiline-resistant cohort, body mass index (BMI) and smear status had one missing data point each, therefore, we used a complete case analysis. For CD4 count, viral load 10% or more were missing (e.g. n=17/57 (30%) and n=17/57 (30%), respectively), and "missing" categories were included. Similarly, an "unknown" category was added for fluoroquinolone resistance in the pooled cohort.

Unfavourable World Health Organization (WHO) treatment outcome was adapted from the WHO 2021 definition[1] with the following differences: 1) treatment failure due lack of sputum culture conversion at six months or reversion at any timepoint was assigned regardless of whether the treatment regimen was changed; and 2) for patients whose regimens were changed due to any baseline drug resistance, outcomes were reported from the time of regimen change, not from treatment start.

We assessed TB-free survival both from the index sputum (bedaquiline-resistant cohort) and from treatment initiation (both cohorts) to account for both programmatic and treatment outcomes, since treatment initiation in the bedaquiline-resistant cohort could be delayed.

Time to death included all deaths during treatment, censored at 18 months, and were assessed both from the index sputum (only the bedaquiline-resistant cohort) and from treatment initiation.

Treatment modification was defined as the addition of two or more drugs from treatment initiation, since no definition of regimen change is included in the latest WHO guideline[2]. The date when the second drug was added was assigned as the date of treatment modification.

For the Cox regression analysis, we tested the proportional hazards assumption using the Schoenfield residuals tests (cox.zph() function from the survival package in RStudio Version 2024·09·0+375 (Posit team, Boston, MA, USA)).

Supplement Table 1: Protocol deviations

Deviation	Justification
Inclusion of a matched control group	Our rationale for including a matched cohort was to (1) provide a measure of internal validity for our findings and (2) contrast treatment-related (culture conversion) and clinical (mortality and TB-free survival) outcome measures with a typical group of patients treated for rifampicin-resistant TB in a national programme.
Addition of time to death analysis	More patients with bedaquiline-resistant TB were identified than originally anticipated, providing more power for an exploratory Cox regression analysis to explore predictors of mortality over 18 months.
Removal of analysis of time to poor clinical outcomes (using the sum of died, lost to follow-up and microbiological failure through 12 and 18 months)	This analysis was not considered relevant as a time endpoint was included in the WHO outcome definition of sputum culture conversion (six months). Additionally, the overall number of patients lost to follow-up using the modified WHO definition was low (n=8), limiting the relevance of conducting an analysis of that group separately. Instead, we conducted an analysis of time to death over 18 months only.
Removal of analysis time to bedaquiline resistance from treatment initiation before the detection of bedaquiline resistance	Since only 23% (n=19/82) of patients had a previous phenotypic drug susceptibility test for bedaquiline showing susceptibility, we did not consider this analysis relevant as we could not ascertain at what time point bedaquiline resistance developed, or if it was present at start of treatment (e.g. primary transmission).
Removal of analysis of time to sputum culture conversion over 6 months	On exploratory data analysis we observed that time to SCC was delayed compared with the matched cohort of patients with bedaquiline-susceptible TB. Extending the time horizon to 12 months enabled us to capture the variability of SCC for the full matched cohort and reduced censoring.

Supplementary Results

The following findings are included following peer review:

- 45/82 (55%) of patients in the bedaquiline-resistant group were on treatment at the time of index sputum collection which had been ongoing for a median of 4·5 months (IQR 1·4, 11·8 months) (Figure 1, Appendix p 6).

- Repeat bedaquiline resistance testing was performed for 30 patients following the index sputum, with a repeat resistant result in 28. Among this group there was a median time to sustained SCC of 307 days (IQR 274, NA) compared with 91 days (IQR 67, 159) for the 54 patients without another test showing bedaquiline resistance. Mortality among those with an additional bedaquiline-resistant isolate was 6/28 (21%) compared with 13/54 (24%) among those who did not have subsequent bedaquiline resistance testing.

- In the first 12 months or until treatment end (whichever came first), patients in the bedaquiline-resistant cohort had a median of 0·7 sputum culture results per month collected (IQR 0·6, 1·0) in routine care. Participants in the prospective SHIFT-TB cohort had study visits aligned with routine care plus two additional study visits for sputum collection (week 2 and week 6); the median number of sputum culture results per month on treatment or until 12 months was 1·0 (IQR 0·8, 1·3). We performed a sensitivity analysis to evaluate the impact of these additional study visits in the bedaquiline-susceptible control cohort on the SCC outputs. After removing the two additional study visits from the control cohort, there was a median of 0·8 sputum culture results per month and time to SCC was 35 days (95% CI 32–42 days), similar to the full analysis, indicating absence of a substantial study protocol effect.

Supplement Table 2: Drug susceptibility testing of sputum and proportion with resistance to each drug before, at, and after the index sputum[1] collection when bedaquiline resistance was identified

**Characteristic**	**Before the index sputum**[1]<br>N = 45[2]	**At the index sputum**[1]<br>N = 82[2]	**After the index sputum**[1]<br>N = 45[2]
**Xpert MTB/Rif Ultra**			
Rifampicin	9/10 (90%)	18/19 (95%)	8/8 (100%)
**Line Probe Assay**			
Rifampicin	44/45 (98%)	72/74 (97%)	42/42 (100%)
Isoniazid	44/45 (98%)	70/74 (95%)	42/43 (98%)
Fluoroquinolone	28/39 (72%)	61/69 (88%)	37/43 (86%)
**Phenotypic DST**			
Amikacin	5/8 (63%)	9/17 (53%)	7/10 (70%)
Bedaquiline	1/20 (5·0%)	82/82 (100%)	28/30 (93%)
Clofazimine	2/11 (18%)	67/73 (92%)	19/21 (90%)
Delaminid	0/0	1/1 (100%)	0/0
Ethambutol	1/1 (100%)	5/7 (71%)	4/5 (80%)
Ethionamide	0/2 (0%)	9/11 (82%)	6/7 (86%)
Isoniazid	3/3 (100%)	10/11 (91%)	5/6 (83%)
Isoniazid, high dose	2/3 (67%)	8/8 (100%)	5/6 (83%)
Levofloxacin	1/6 (17%)	15/22 (68%)	7/11 (64%)
Linezolid	2/28 (7·1%)	2/77 (2·6%)	7/33 (21%)
Moxifloxacin	12/14 (86%)	24/26 (92%)	8/10 (80%)
Moxifloxacin, high dose	12/15 (80%)	21/32 (66%)	7/13 (54%)
Para-amino salicylic acid	0/2 (0%)	0/12 (0%)	2/9 (22%)
Pyrazinamide	1/3 (33%)	4/5 (80%)	5/6 (83%)
Rifabutin	1/2 (50%)	9/13 (69%)	8/10 (80%)

[1]Index sputum is defined as the first bedaquiline-resistant *Mycobacterium tuberculosis* isolate

[2]n/N (%), DST = Drug susceptibility testing

Supplement Table 3: Treatment regimens and treatment duration from treatment initiation[1] after the index sputum during which bedaquiline resistance was identified

Characteristic	Before index sputum[2]<br>N = 45[3]	After index sputum[2]<br>N = 82[3]
Bedaquiline	43 (96%)	72 (88%)
Clofazimine	45 (100%)	79 (96%)
Linezolid	42 (93%)	79 (96%)
Terizidone	36 (80%)	78 (95%)
Delamanid	18 (40%)	61 (74%)
Para-aminosalicylic acid	18 (40%)	61 (74%)
Levofloxacin	35 (78%)	53 (65%)
Pyrazinamide	28 (62%)	43 (52%)
Meropenem (plus amoxicillin-clavulanate)	3 (6·7%)	32 (39%)
Isoniazid	18 (40%)	27 (33%)
Ethambutol	21 (47%)	25 (30%)
Moxifloxacin	3 (6·7%)	14 (17%)
Ethionamide	5 (11%)	6 (7·3%)
Rifabutin	2 (4·4%)	5 (6·1%)
Amikacin	0 (0%)	0 (0%)
Kanamycin	3 (6·7%)	0 (0%)
Time to meropenem initiation after index sputum collection, days	NA	156 (115, 236) (1, 576)
Time to meropenem initiation after treatment start, days	NA	140 (45, 213) (0, 576)
Duration of meropenem treatment, days	32 (4, 181) (4, 181)	171 (135, 199) (32, 457)
Duration of linezolid treatment, days	82 (16, 153) (1, 574)	391 (173, 583) (4, 858)
Time of bedaquiline treatment, days	120 (30, 169) (1, 380)	167 (94, 215) (4, 413)
Treatment duration, days	121 (31, 251) (1, 819)	539 (317, 620) (4, 1379)
Treatment modification[4]	NA	44 (54%)

[1]Treatment initiation following the index sputum.

[2]Index sputum is defined as the first bedaquiline-resistant *Mycobacterium tuberculosis* isolate

[3]n (%); Median (Q1, Q3) (Min, Max)

[4]Defined as the addition of at least two new drugs after the index sputum

NA = Not applicable

Supplement Table 4: Sustained sputum culture conversion over 12 months after treatment initiation[1] in patients with bedaquiline-resistant tuberculosis using a Cox proportional hazards model

Characteristic	Number of events	HR	95% CI	p-value
Age	50	1·01	0·99, 1·03	0·26
Sex, healthcare worker reported				
Male	24	··	··	
Female	26	1·02	0·59, 1·78	0·93
BMI	49	1·04	0·98, 1·10	0·22
HIV				
No	13	··	··	
Yes	37	1·43	0·76, 2·69	0·27
Combined HIV and ART status				
HIV negative	13	··	··	
HIV positive, on ART	34	1·48	0·78, 2·80	0·23
HIV positive, not on ART	3	1·05	0·30, 3·67	0·94
Previous RR-TB				
No	24	··	··	
Yes	26	1·03	0·59, 1·80	0·90
Baseline microscopy status				
Negative	25	··	··	
Positive	25	0·95	0·54, 1·66	0·86
Bedaquiline use				
No	4	··	··	
Yes	46	2·02	0·73, 5·63	0·18

[1]Treatment initiation following the index sputum. Index sputum is defined as the first bedaquiline-resistant *Mycobacterium tuberculosis* isolate

ART = Antiretroviral therapy, BMI = Body Mass Index, CI = Confidence Interval, Human Immunodeficiency Virus, HR = Hazard Ratio, RR-TB = Rifampicin-rRsistant tuberculosis

Censored at death, lost to follow-up, and 12 months

Supplement Table 5: Time to sustained sputum culture conversion over 12 months after treatment initiation in bedaquiline-resistant cases and matched bedaquiline-susceptible controls using a stratified Cox proportional hazard model

	Univariable Analysis				Multivariable Analysis		
**Characteristic**	**Number of events**	**HR**	**95% CI**	**p-value**	**HR**	**95% CI**	**p-value**
Sex, healthcare worker reported							
Male	63	··	··				
Female	59	1·07	0·52, 2·22	0·85			
BMI	121	0·98	0·91, 1·06	0·62			
Baseline microscopy grade	122	0·72	0·56, 0·92	0·0081	0·42	0·23, 0·76	0·005
Baseline fluoroquinolone resistance							
No	56	··	··				
Yes	17	0·21	0·10, 0·44	<0·0001	3·41	0·28, 40·8	0·33
Unknown	49	1·54	0·39, 6·16	0·54	5·68	0·27, 121	0·27
Bedaquiline resistance							
No	72	··	··		··	··	
Yes	50	0·16	0·08, 0·31	<0·0001	0·03	0·0023, 0·29	0·003

BMI = Body Mass Index, HR = Hazard Ratio, CI = Confidence Interval

Censored at death, lost to follow-up, and 12 months

Supplement Table 6: Modified World Health Organization treatment outcomes after treatment initiation[1] in patients with bedaquiline-resistant tuberculosis

**World Health Organization Treatment Outcome**	**N = 81** [2,3]
Favourable treatment outcome	27 (33%)
Cured	24 (30%)
Treatment completed	3 (3·7%)
Unfavourable treatment outcome	54 (67%)
Died	11 (14%)
Lost to follow-up	8 (9·9%)
Treatment failed	35 (43%)
Lack of culture conversion by month 6	14/35 (40%)
Culture reversion	10/35 (29%)
Change of regimen and/or permanently changed ≥2 drugs	11/35 (31%)
Poor clinical response and/or no bacteriological response based on clinical judgement	11/11 (100%)

[1]Treatment initiation following the index sputum. Index sputum is defined as the first bedaquiline-resistant *Mycobacterium tuberculosis* isolate

[2]n (%)

[3]One patient was not yet assigned a World Health Organization outcome due to ongoing treatment at the end of the study (20 January 2025) and was excluded from this analysis.

Supplement Table 7: Tuberculosis-free survival at 6, 12, and 18 months after treatment initiation[1] in patients with bedaquiline-resistant tuberculosis

Characteristic	Month 6<br>N = 82[2]	Month 12<br>N = 82[2]	Month 18<br>N = 81[2, 3]
Achieved	45 (55%)	47 (57%)	41 (51%)
Alive, TB free & treatment complete[4]	0 (0%)	2 (2·4%)	8 (9·8%)
Alive, TB free & in care (treatment ongoing)	45 (55%)	45 (55%)	33 (41%)
Not achieved	37 (45%)	35 (43%)	40 (49%)
Not alive (i.e. died)	8 (9·8%)	13 (16%)	19 (23%)
Not TB free, alive & in care (treatment ongoing)	23 (28%)	18 (22%)	16 (20%)
Not in care[5]	6 (7·3%)	4 (4·9%)	5 (6·1%)
Alive, TB free	2 (2·4%)	2 (2·4%)	3 (3·7%)
Alive, not TB free	4 (4·9%)	2 (2·4%)	2 (2·4%)

[1]Treatment initiation following the index sputum. Index sputum is defined as the first bedaquiline-resistant *Mycobacterium tuberculosis* isolate

[2]n (%)

[3]18 months post-treatment initiation had not yet passed for one participant at the date of database closure

[4]TB-free is defined as achieving sputum culture conversion without culture reversion by the specified timepoint.

[5]Lost to follow up (irrespective of TB status) or completed treatment but not TB-free.

TB = Tuberculosis

Supplement Table 8: Unfavourable tuberculosis-free survival at 18 months after treatment initiation in patients with bedaquiline-resistant tuberculosis using a logistic regression model

			Univariable analysis			Multivariable analysis		
	Unfavourable TB-free survival[1]	Favourable TB-free survival[1]	OR	95% CI	p-value	OR	95% CI	p-value
Age (years)	36·0 (28·5, 44·5)	39·0 (33·0, 39·4)	0·98	0·94, 1·01	0·22			
Sex, healthcare worker reported								
Female	20/42 (48%)	22/42 (52%)	—	—				
Male	19/40 (48%)	21/40 (53%)	1·00	0·42, 2·38	>0·99			
Body Mass Index	17·6 (16·05, 21·35)	19·4 (17·0, 22·6)	0·94	0·85, 1·03	0·20			
HIV status								
HIV infection	25/57 (44%)	32/57 (56%)	—	—				
No HIV infection	14/25 (56%)	11/25 (44%)	1·63	0·63, 4·27	0·31			
Combined HIV and ART Status								
HIV negative	14/25 (56%)	11/25 (44%)	—	—				
HIV positive, not on ART	2/6 (33%)	4/6 (67%)	0·39	0·05, 2·40	0·33			
HIV positive, on ART	23/51 (45%)	28/51 (55%)	0·65	0·24, 1·68	0·37			
Baseline microscopy status								
Negative	15/38 (39%)	23/38 (61%)	—	—				
Positive	24/44 (55%)	20/44 (45%)	1·84	0·77, 4·50	0·17			
Previous RR-TB								
No	19/41 (46%)	22/41 (54%)	—	—				
Yes	20/41 (49%)	21/41 (51%)	1·10	0·46, 2·64	0·83			
Number of medications at initiation	6 (5, 7)	7 (5, 7)	0·67	0·44, 0·98	0·044	0·66	0·43, 0·97	0·042
Bedaquiline duration (months)	4 (1, 6)	6 (3·5, 7)	0·89	0·76, 1·02	0·10	0·88	0·75, 1·02	0·10
Meropenem								
No	22/51 (43%)	29/51 (57%)	—	—				
Yes	17/31 (55%)	14/31 (45%)	1·60	0·65, 3·98	0·30			
P-aminosalicylic acid								
No	12/21 (57%)	9/21 (43%)	—	—				
Yes	27/61 (44%)	34/61 (56%)	0·60	0·21, 1·61	0·31			
Delamanid								
No	7/21 (33%)	14/21 (67%)	—	—				
Yes	32/61 (52%)	29/61 (48%)	2·21	0·80, 6·54	0·13			
Pyrazinamide								
No	20/50 (40%)	30/50 (60%)	—	—				
Yes	19/42 (45%)	23/42 (55%)	0·83	0·34, 1·97	0·67			
Isoniazid								
No	26/55 (47%)	29/55 (53%)	—	—				
Yes	13/27 (48%)	14/27 (52%)	1·04	0·41, 2·61	0·94			
Fluoroquinolone								
No	13/24 (54%)	11/24 (46%)	—	—				
Yes	26/58 (45%)	32/58 (55%)	0·69	0·26, 1·79	0·44			
Ethionamide								
No	26/57 (46%)	31/57 (54%)	—	—				
Yes	13/25 (52%)	12/27 (44%)	1·29	0·50, 3·35	0·59			

[1]Median (Q1, Q3) (Min, Max); n/N (%)

ART = Antiretroviral Therapy, CI = Confidence Interval, HIV = Human Immunodeficiency Virus, OR = Odds Ratio, RR-TB = Rifampicin-Resistant Tuberculosis

Supplement Table 9: Tuberculosis-free survival at 6, 12, and 18 months after treatment start in the control group with bedaquiline-susceptible tuberculosis

**Characteristic**	**Month 6** N = 82[1]	**Month 12** N = 82[1]	**Month 18** N = 82[1]
Achieved	64 (78%)	56 (71%)	48 (63%)
Alive, TB free & treatment complete[2]	0 (0%)	34 (41%)	32 (39%)
Alive, TB free & in care (treatment ongoing) [2]	64 (78%)	22 (27%)	16 (20%)
Not achieved	18 (22%)	23 (29%)	28 (37%)
NOT alive (i.e. died)	7 (8·5%)	9 (11%)	15 (18%)
NOT TB free, alive & in care (treatment ongoing)	4 (4·9%)	3 (3·7%)	2 (2·4%)
Not in care[3]	7 (8·5%)	11 (13%)	11 (13%)
Alive, TB free[2]	7 (8·5%)	10 (12%)	11 (13%)
Alive, not TB free[2]	0 (0%)	1 (1·2%)	0 (0%)
Not assessable	0 (0%)	3 (3·7%)	6 (7·3%)

[1]n (%)

[2]TB-free is defined as achieving sputum culture conversion without culture reversion by the specified timepoint.

[3]Lost to follow up (irrespective of TB status) or completed treatment but not TB-free.

TB = Tuberculosis

Supplement Table 10: Time to death over 18 months from index sputum[1] collection in patients with bedaquiline-resistant tuberculosis using a Cox proportional hazards model

Characteristic	Univariable Analysis				Multivariable analysis		
	Number of events	HR	95% CI	p-value	HR	95% CI	p-value
Age	19	1·01	0·97, 1·05	0·65			
Sex, healthcare worker reported							
Male	10	—	—				
Female	9	0·83	0·34, 2·03	0·68			
BMI	19	0·88	0·77, 1·01	0·069	0·89	0·78, 1·02	0·10
HIV							
No	6	—	—				
Yes	13	0·93	0·35, 2·44	0·88			
Combined HIV and ART							
HIV negative	6	—	—				
HIV positive, on ART	11	0·87	0·32, 2·36	0·79			
HIV positive, not on ART	2	1·41	0·28, 6·98	0·67			
Baseline microscopy							
Negative	7	—	—				
Positive	12	1·58	0·62, 4·02	0·33			
Previous RR-TB							
No	10	—	—				
Yes	9	0·90	0·37, 2·22	0·82			
CD4 count							
< 200	5	—	—				
≥ 200	4	0·89	0·24, 3·30	0·86			
Unknown	4	0·95	0·25, 3·53	0·93			
Duration of bedaquiline (months)	19	0·73	0·61, 0·87	0·0006	0·74	0·62, 0·88	0·0008

[1]Index sputum is defined as the first bedaquiline-resistant *Mycobacterium tuberculosis* isolate

BMI = Body Mass Index, CI = Confidence Interval, HIV = Human Immunodeficiency Virus, HR = Hazard Ratio, RR-TB = Rifampicin-Resistant Tuberculosis

Censored at 18 months.

Supplement Table 11: Time to death over 18 months from index sputum[1] in patients with bedaquiline-resistant tuberculosis who survived at least eight weeks using a Cox proportional hazards model (sensitivity analysis)

	Univariable Analysis				Multivariable Analysis		
**Characteristic**	**Number of events**	**HR**	**95% CI**	**p-value**	**HR**	**95% CI**	**p-value**
Age	15	1·02	0·98, 1·06	0·33			
Sex, healthcare worker reported							
Male	7	—	—				
Female	8	1·04	0·38, 2·87	0·94			
BMI	15	0·83	0·70, 0·99	0·040	0·84	0·71, 1·00	0·053
HIV							
No	5	—	—				
Yes	10	0·84	0·29, 2·47	0·77			
Combined HIV and ART							
HIV negative	5	—	—				
HIV positive, on ART	8	0·75	0·24, 2·29	0·61			
HIV positive, not on ART	2	1·70	0·33, 8·76	0·53			
Baseline microscopy							
Negative	6	—	—				
Positive	9	1·40	0·50, 3·94	0·52			
Previous RR-TB							
No	9	—	—				
Yes	6	0·66	0·23, 1·86	0·43			
CD4 count							
< 200	3	—	—				
≥ 200	4	1·44	0·32, 6·45	0·63			
Unknown	3	1·15	0·23, 5·70	0·86			
Duration of bedaquiline (months)	15	0·80	0·66, 0·96	0·018	0·81	0·68, 0·97	0·022

[1]Index sputum is defined as the first bedaquiline-resistant *Mycobacterium tuberculosis* isolate

ART = Antiretroviral Therapy, CI = Confidence Interval, HIV = Human Immunodeficiency Virus, HR = Hazard Ratio, RR-TB = Rifampicin-Resistant Tuberculosis

Censored at 18 months.

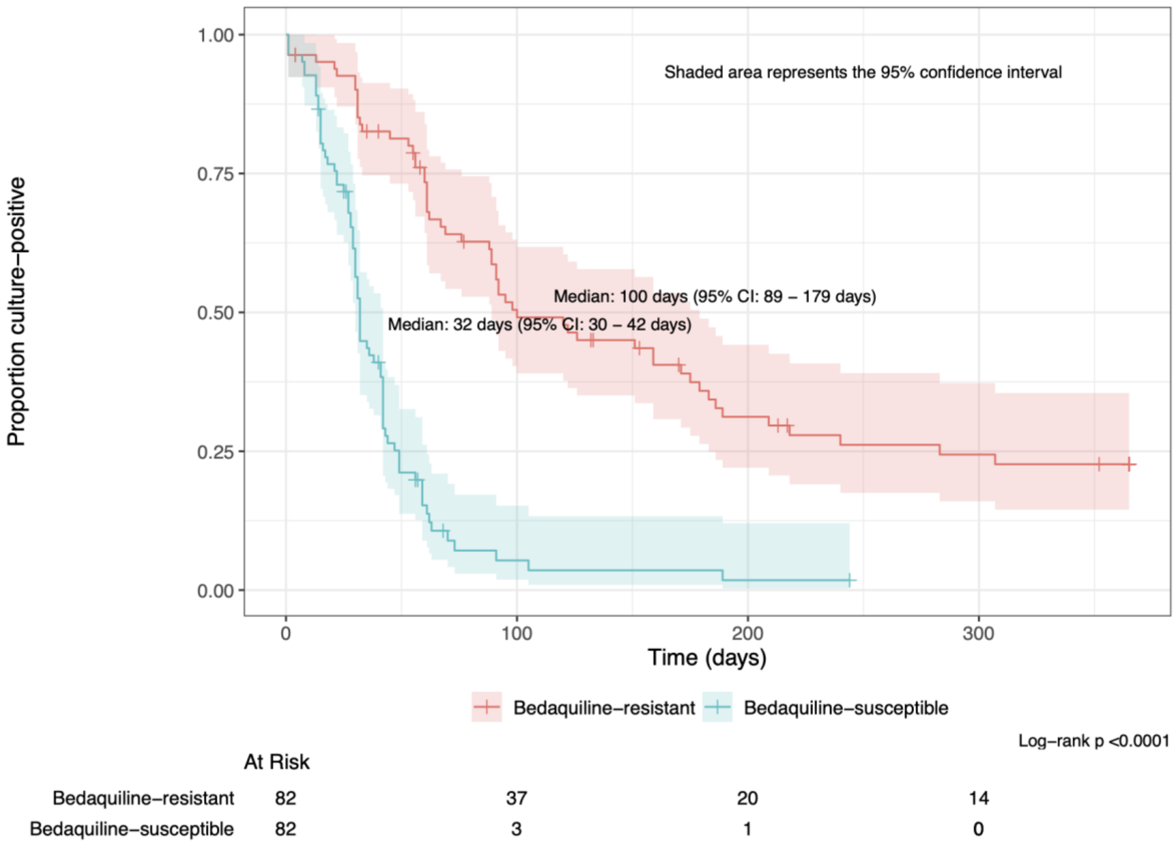

Supplement Figure 1: Time to first sputum culture conversion from treatment initiation in bedaquiline-resistant cases and matched bedaquiline-susceptible controls until 12 months, by bedaquiline resistance

Censored at death, lost to follow-up, and at 12 months

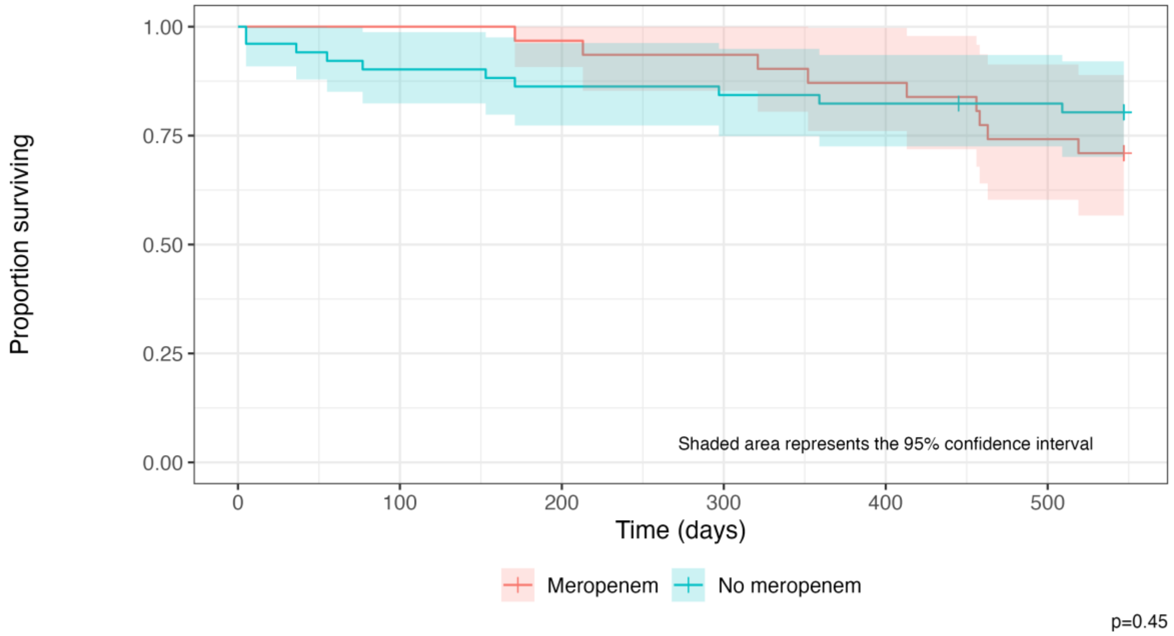

At Risk

Meropenem	31	31	30	29	27	23
No meropenem	51	46	44	43	42	41

Supplement Figure 2: Survival from time of index sputum[1] collection in patients with bedaquiline resistant tuberculosis until 18 months, by meropenem use

[1]Index sputum is defined as the first bedaquiline-resistant *Mycobacterium tuberculosis* isolate

Censored at 18 months

References

1. World Health Organization. WHO operational handbook on tuberculosis. Module 4: Treatment - drug-resistant tuberculosis treatment, 2022 update. Geneva: WHO, 2022. https://www.who.int/publications/i/item/9789240065116 (accessed 10 November 2024).

2. World Health Organization. WHO consolidated guidelines on tuberculosis. Module 4: treatment - drug-resistant tuberculosis treatment, 2022 update. 2022. https://www.who.int/publications/i/item/9789240007048 (accessed 10 November 2024).
